# Master–Slave Outer Synchronization in Different Inner–Outer Coupling Network Topologies

**DOI:** 10.3390/e25050707

**Published:** 2023-04-24

**Authors:** Adrian Arellano-Delgado, Rosa Martha López-Gutiérrez, Miguel Ángel Murillo-Escobar, Cornelio Posadas-Castillo

**Affiliations:** 1 National Council of Science and Technology, Ciudad de Mexico 03940, CDMX, Mexico; 2 Engineering, Architecture and Design Faculty, Autonomous University of Baja California, Ensenada 22860, BC, Mexico; 3 Electronics and Telecommunication Department, Scientific Research and Advanced Studies Center of Ensenada, Ensenada 22860, BC, Mexico; 4 Facultad de Ingeniería Mecánica y Eléctrica, Universidad Autónoma de Nuevo León, San Nicolás de los Garza 66455, NL, Mexico

**Keywords:** inner–outer network topology, outer synchronization, master stability function, diffusive coupling, MACM chaotic system

## Abstract

In this work, the problem of master–slave outer synchronization in different inner–outer network topologies is presented. Specifically, the studied inner–outer network topologies are coupled in master–slave configuration, where some particular scenarios concerning inner–outer topologies are addressed in order to disclose a suitable coupling strength to achieve outer synchronization. The novel MACM chaotic system is used as a node in the coupled networks, which presents robustness in its bifurcation parameters. Extensive numerical simulations are presented where the stability of the inner–outer network topologies is analyzed through a master stability function approach.

## 1. Introduction

The emergence of rhythmic behaviors, such as synchronization, is a phenomenon that can occur in many areas of daily life. Specifically, the phenomenon of synchronization, i.e., the temporal adjustment of events between two or more objects or subjects, see [1], is extensively studied by the scientific community today, and much of the effort is devoted to analyzing how to achieve synchronization in networks. The phenomenon of synchronization is found in multiple scientific areas, such as physics, chemistry, mathematics, and computer science, see [2,3,4,5,6,7,8,9]. From the point of view of graph theory [10], we can establish two of the main causes that directly affect achieving synchronization, (i) how the nodes are communicated and (ii) how the nodes are coupled, where the first cause can be divided in master–slave configuration (unidirectionally) and/or mutual configuration (bidirectionally), see [1,11], and the second cause could be divided into inner and/or outer coupling, that is the inner and outer connection topology, see [12,13,14,15,16,17,18,19,20,21] for the respective coupling.

In many recent works, the synchronization problem is addressed for different, or even combinations, of the aforementioned cases, where the case of outer synchronization is taken as a more general case. We can cite some examples: in [22], the authors present an outer coupling to achieve exponential synchronization between two networks, on the other hand, in [23], the authors analyze an outer coupling for two fractional-order networks in a master–slave configuration, in addition, outer synchronization between two complex dynamical networks with discontinuous coupling is analyzed in [24], and another recent work, see [25], presents outer coupling to achieve synchronization between delayed coupling networks with uncertain parameters, demonstrating the versatility of the study about outer coupling for networks synchronization.

As we can see, many recent works have focused on analyzing outer synchronization, disclosing that it is a topic of current interest. On the other hand, in most of the aforementioned works, the effect of combining the different cases of communication and coupling among nodes in a network is not analyzed, which gives rise to the conception of this work, which focuses on addressing the analysis of different ways to couple and communicate nodes in networks. Particularly, in this work, we focus on analyzing synchronization for different inner–outer network topologies in master–slave configuration.

This work is organized as follows. In Section 2, details of how the analyzed networks are built and also some preliminaries of synchronization of complex networks are presented. In Section 3, we present the master stability function approach to study and compare the stability of the synchronization state of the analyzed networks. In Section 4, the MACM chaotic system and its characteristics are described. In Section 5, we present the main results from the different analyzed inner–outer network topologies, and a numerical example for a large number of networks is also presented. Finally, some conclusions are drawn in Section 6.

## 2. Preliminaries in Complex Dynamical Networks

In this section, we give some preliminaries of complex networks, inner–outer coupling network topologies, and synchronization. In this work, we consider *M* networks composed of *N* nodes. The inner–outer coupling network topologies are represented by $A_{\mathrm{in}}$ and $A_{\mathrm{out}}$, respectively, which gives rise to the emergence of a complex network of networks of M×N nodes. The communication of the nodes is made in a master–slave configuration both for inner and outer networks, where each node constitutes a *n*-dimensional chaotic dynamical system, described as follows

(1) $${\dot{x}}_{i}=f\left(x_{i}\right)+u_{i},$$

with i=1,2,…,M×N, where $x_{i}={\left(x_{i,1},\;x_{i,2},\;\ldots ,\;x_{i,n}\right)}^{T}\in R^{n}$ is the state vector of the node *i* and $u_{i}={\left(u_{i,1},u_{i,2},\ldots ,u_{i,n}\right)}^{T}\in R^{n}$ is the input signal of the node *i*. Moreover, a diffusive coupling that is well-known and extensively studied is used as follows,

(2) $$u_{i}=\left(A⊗\Gamma \right)x_{i}$$

where $\Gamma_{n\times n}$ is a constant 0 or 1 matrix that determines the selection of the state variables used in the inner and outer couplings, $A_{\left(M\times N)\times (M\times N\right)}$ is the coupling matrix of the entire network described as follows,

(3) $$A=c_{1}A_{\mathrm{in}}+c_{2}A_{\mathrm{out}}=c_{1}\left(I⊗A_{i}\right)+c_{2}\left(A_{o}⊗\Gamma_{o}\right)$$

where $A_{\mathrm{in}(M\times N)\times (M\times N)}$ and $A_{\mathrm{out}(M\times N)\times (M\times N)}$ are the inner and outer coupling matrices respectively; $c_{1}$ and $c_{2}$ are the inner and outer coupling strengths, respectively; $I_{\left(M\times M\right)}$ is the identity matrix; $\Gamma_{o(N\times N)}$ is a constant 0 or 1 diagonal matrix that determines the node selection to be used in order to couple the networks (note that if we want to use cross nodes in the outer coupling, we must add the corresponding term in (3) for this type of links); $A_{i}$ and $A_{o}$ are suitable base matrices of inner and outer coupling topologies, where ⊗ is the Kronecker product.

**Figure 1:**
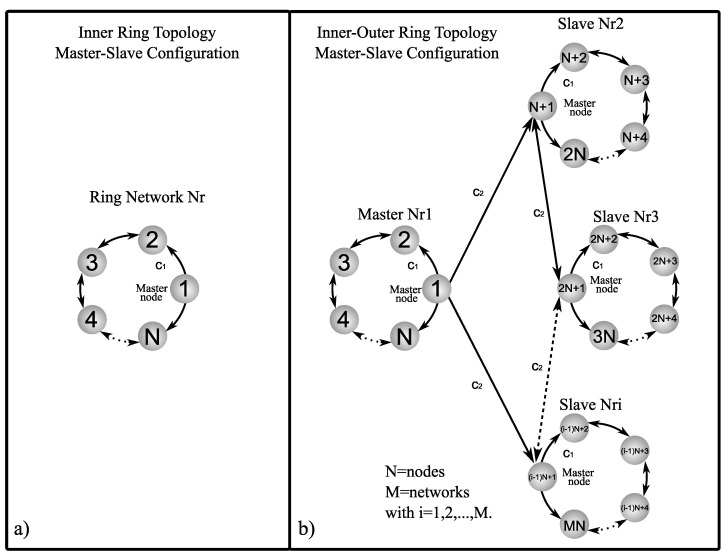
Graphic representation of ring network: (**a**) inner ring topology in master–slave configuration and (**b**) inner–outer ring coupling topology in master–slave configuration.

**Figure 2:**
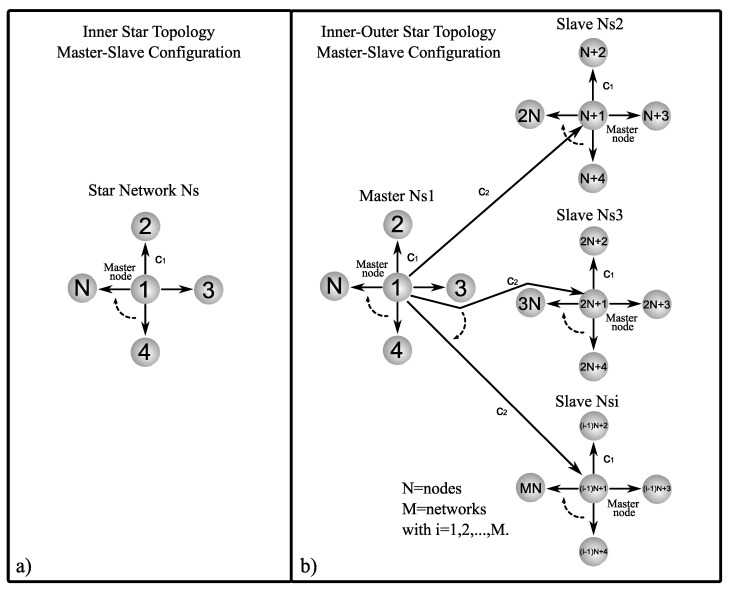
Graphic representation of star network: (**a**) inner star topology in master–slave configuration and (**b**) inner–outer star coupling topology in master–slave configuration.

**Figure 3:**
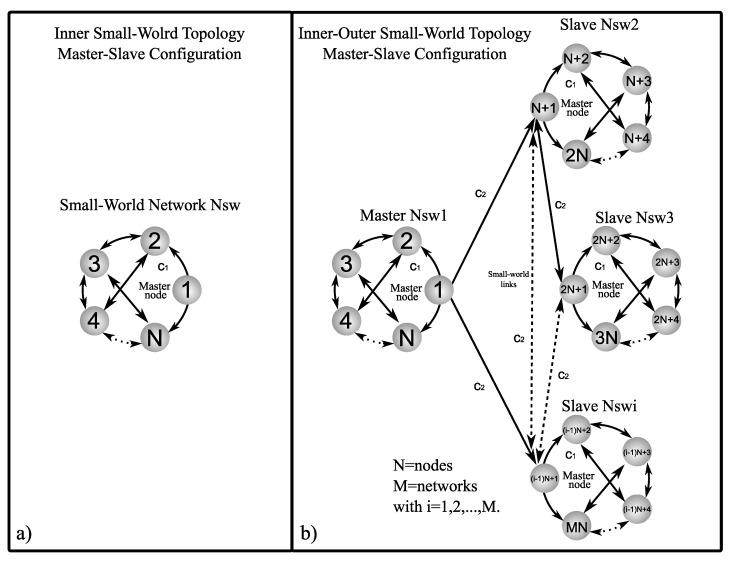
Graphic representation of small-world network: (**a**) inner small-world topology in master–slave configuration and (**b**) inner–outer small-world coupling topology in master–slave configuration.

Now, suppose we have connected complex networks, it can be shown that zero is an eigenvalue of A with multiplicity 1 and all the other eigenvalues are strictly negative, see [26,27]. Figure 1, Figure 2 and Figure 3 show a graphic representation of the general scheme of the inner–outer coupling network topologies to be used in this work.

According to [27], the complex networks (1) is said to achieve (asymptotically) synchronization, if:

(4) $$x_{1}\left(t\right)=x_{2}\left(t\right)=\;\ldots \;=x_{M\times N}\left(t\right),\;\;as\;\;t\to \infty .$$

It is desired that coupling conditions (2) and (3) guarantee that the synchronization state be a solution, $x_{1}\left(t\right)\in R^{n}$, of the master node of the master network, that is

(5) $${\dot{x}}_{1}\left(t\right)=f\left(x_{1}\left(t\right)\right),$$

where $x_{1}\left(t\right)$ can be an equilibrium point, a periodic orbit, or a chaotic attractor. Thus, the stability of the synchronization state,

(6) $$x_{2}\left(t\right)=x_{3}\left(t\right)=\;\ldots \;=x_{M\times N}\left(t\right)=x_{1}\left(t\right),$$

of the complex network of networks (1) is determined by the dynamics of the master chaotic node $x_{1}\left(t\right)$, matrix Γ, and the coupling matrix A (with their respective implied coupling strengths $c_{1}$, $c_{2}$, and matrices $A_{i}$, $A_{o}$, and $\Gamma_{o}$).

## 3. Master Stability Function

We use the master stability function to study and compare the stability of inner and outer network synchronization [28]. According to [28], for (2) and (3), each block of the diagonalized variational equation by blocks is as follows

(7) $${\overset{\cdot }{\xi }}_{k}=\left[Df\left(x_{1}\right)+\zeta_{k}\Gamma \right]\xi_{k}$$

with k=0,1,2,…,(M×N)−1, where $\zeta_{k}$ is an eigenvalue of the coupling matrix A, with $\zeta_{0}=0$. As mentioned in Section 2, Γ determines the state variables to be used in the couplings, where the maximum Lyapunov exponent $\lambda_{max}$ is calculated for the generic variational Equation (7). By using certain inner and outer coupling strengths $c_{1}$ and $c_{2}$, the sign of $\lambda_{max}$ is verified, which indicates the synchronization state, for $\lambda_{max}<0$ the synchronization state is stable, while for $\lambda_{max}>0$, the synchronization state is unstable. For the computational calculation of the maximum Lyapunov exponents $\lambda_{max}$, we use a modified version of the algorithm presented in [29]. For the calculation of the maximum Lyapunov exponents, we use the programming software Matlab with initial conditions $\;x_{1}\left(0\right)={\left[0.1,0.1,0.1\right]}^{T}$ in the Ode45 function for a simulation of 100 time units.

## 4. MACM Chaotic System

**Figure 4:**
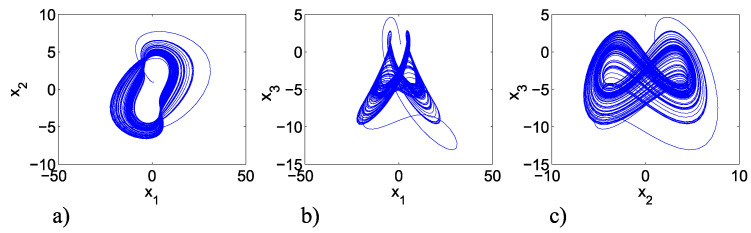
Phase planes of the MACM chaotic system (8) with a=2, b=2, c=0.5, and d=10: (**a**) $x_{1}$ versus $x_{2}$ phase plane; (**b**) $x_{1}$ versus $x_{3}$ phase plane; (**c**) $x_{2}$ versus $x_{3}$ phase plane.

In this section, we present the chaotic MACM system used as a node that has robustness in its bifurcation parameters, see [30]. The chaotic system is given by

(8) $$\left[\begin{array}{c} {\dot{x}}_{1}\\ {\dot{x}}_{2}\\ {\dot{x}}_{3} \end{array}\right]=\left[\begin{array}{c} -ax_{1}-bx_{2}x_{3}\\ -x_{1}+cx_{2}\\ d-x_{2}^{2}-x_{3} \end{array}\right]$$

where *a*, *b*, *c*, *d* $\in R^{+}$ and $x_{1}={\left(x_{1},\;x_{2},\;x_{3}\right)}^{T}\in R^{n}$ is the state vector of the system (8). Figure 4 shows the attractors when parameters values are a=b=2, c=0.5, and d=10, and initial conditions are $\left(x_{1},x_{2},x_{3}\right)=\left(1,1,1\right)$.

**Figure 5:**
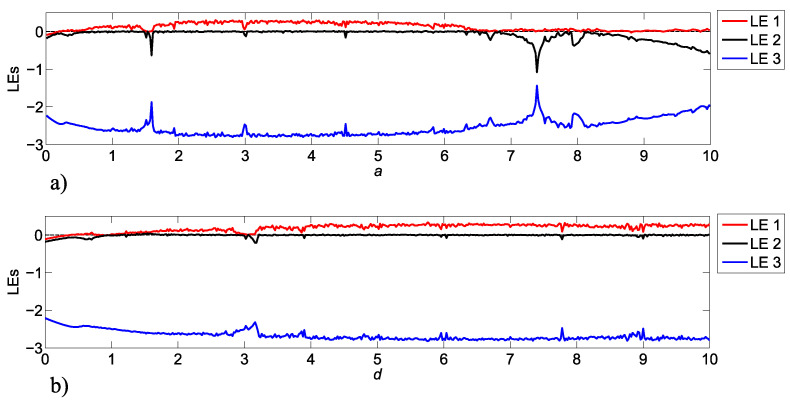
LEs for MACM chaotic system (8) with b=2 and c=0.5 for: (**a**) 0≤a≤10 and (**b**) 0≤b≤10 .

The robustness of bifurcation parameter values makes the MACM system (8) suitable to be used in different applications, for example, in secure message transmission. The Lyapunov exponents (LEs) for a variation of the parameter bifurcation values *a* and *d* in a range from 0 to 10 are shown in Figure 5.

Note that there are parameter values that can generate instability in system (8), for example, if we use 0<a<0.8, system (8) becomes unstable, so it is important to select parameter values (see Figure 5) that generate chaotic attractors.

## 5. Analysis of Master–Slave Inner–Outer Coupling Network Topologies

This section addresses the analysis and comparison of different scenarios involving master–slave inner and outer coupled networks in different topologies. The coupled nodes within the inner–outer coupled network are described as follows

(9) $$\left[\begin{array}{c} {\dot{x}}_{i1}\\ {\dot{x}}_{i2}\\ {\dot{x}}_{i3} \end{array}\right]=\left[\begin{array}{c} -ax_{i1}-bx_{i2}x_{i3}+u_{i1}\\ -x_{i1}+cx_{i2}+u_{i2}\\ d-x_{i2}^{2}-x_{i3}+u_{i3} \end{array}\right],$$

where i=1,2,…,M×N.

It is important to note that when we use parameter values a=b=2, c=0.5, d=10, and initial conditions $\left(x_{11},x_{12},x_{13}\right)=\left(1,1,1\right)$, that is, in the master node, a chaotic motion (chaotic attractor) is established for the synchronization state in (6).

### 5.1. Inner Topology of Ring, Star, and Small-World Networks in Master–Slave Configuration

**Figure 6:**
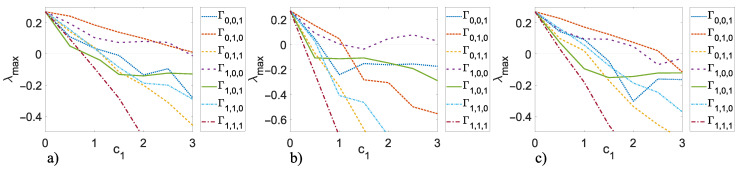
Maximum Lyapunov exponent $\lambda_{max}$ for $0\leq c_{1}\leq 3$ and different values of Γ: (**a**) inner ring topology, (**b**) inner star topology, and (**c**) inner small-world topology.

First of all, inner coupled networks in ring, star, and small-world topologies are analyzed, where the coupling matrices $A_{i}$ corresponding to the different coupling topologies in master–slave configuration are given as follows, for the inner ring topology (see Figure 1a) the matrix $A_{i}$ is

(10) $$A_{i}=A_{\mathrm{ir}}=\left[\begin{array}{ccccc} 0 & 0 & 0 & 0 & 0\\ 1 & -2 & 1 & 0 & 0\\ 0 & 1 & -2 & 1 & 0\\ 0 & 0 & 1 & -2 & 1\\ 1 & 0 & 0 & 1 & -2 \end{array}\right],$$

where the sub-index r indicates ring, while for the inner star topology (see Figure 2a) the matrix $A_{i}$ is defined as follows

(11) $$A_{i}=A_{\mathrm{is}}=\left[\begin{array}{ccccc} 0 & 0 & 0 & 0 & 0\\ 1 & -1 & 0 & 0 & 0\\ 1 & 0 & -1 & 0 & 0\\ 1 & 0 & 0 & -1 & 0\\ 1 & 0 & 0 & 0 & -1 \end{array}\right],$$

where the sub-index s indicates star. An interesting type of non-regular network is the so-called small-world network. These types of networks, which are neither regular nor random, are found mostly in technological, biological, and social networks. In this work, we use the model of Newman and Watts (see [31,32]) in order to build a very simple small-world network to use in our analysis. We started with a ring topology network to which links are added with a probability $p_{1}=0.3$ obtaining a clustering coefficient of 0.4, an average path length of 1.04, and an average grade of 2.8. The resulting matrix for the inner small-world topology (see Figure 3a)) is as follows

(12) $$A_{i}=A_{\mathrm{isw}}=\left[\begin{array}{ccccc} 0 & 0 & 0 & 0 & 0\\ 1 & -3 & 1 & 1 & 0\\ 0 & 1 & -3 & 1 & 1\\ 0 & 1 & 1 & -3 & 1\\ 1 & 0 & 1 & 1 & -3 \end{array}\right],$$

where the sub-index sw indicates small-world. In order to establish which state variable is better to use in the master–slave communication among the nodes of the networks, we calculate the maximum Lyapunov exponent $\lambda_{max}$ in (1) using (8) as a node and applying (10)–(12) with different values (the most representative) of Γ as follows: $\Gamma_{0,0,1}=diag\left[0,0,1\right]$, $\Gamma_{0,1,0}=diag\left[0,1,0\right]$, $\Gamma_{0,1,1}=diag\left[0,1,1\right]$, $\Gamma_{1,0,0}=diag\left[1,0,0\right]$, $\Gamma_{1,0,1}=diag\left[1,0,1\right]$, $\Gamma_{1,1,0}=diag\left[1,1,0\right]$, and $\Gamma_{1,1,1}=diag\left[1,1,1\right]$. Figure 6 shows the $\lambda_{max}$ of the different values of Γ used in the ring, star, and small-world inner coupling topologies. Note that for the results shown in Figure 6, $c_{2}$ in (3) is zero and $\lambda_{max}$ is obtained for $0\leq c_{1}\leq 3$ with steps of 0.5. Consequently, we can deduce that $\Gamma_{1,0,1}=diag\left[1,0,1\right]$ is a suitable value to couple the nodes because if we use only two state variables in order to couple the nodes, the best result is obtained, therefore, we propose to use $\Gamma_{1,0,1}$ in the analyzed topologies in order to achieve inner and outer synchronization. Note that in case of carrying out the numerical simulations to corroborate the results in Figure 6, possible values of initial conditions are $x_{i1,i2,i3}\left(0\right)=\left[rand\left(1.01,1.02\right),0.1,0.1\right]$ for i=1,2,…,N×M.

### 5.2. Outer Topology of Ring, Star, and Small-World Networks in Master–Slave Configuration

**Figure 7:**
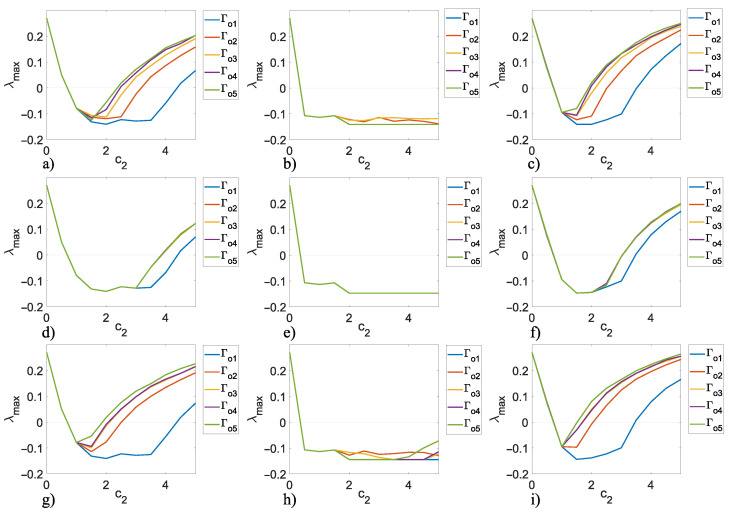
Maximum Lyapunov exponent $\lambda_{max}$ for $c_{1}=2$, $0\leq c_{2}\leq 5$ and $\Gamma_{o1}=diag\left[1,0,0,0,0\right]$, $\Gamma_{o2}=diag\left[1,1,0,0,0\right]$, $\Gamma_{o3}=diag\left[1,1,1,0,0\right]$, $\Gamma_{o4}=diag\left[1,1,1,1,0\right]$, $\Gamma_{o5}=diag\left[1,1,1,1,1\right]$: (**a**) R−R, (**b**) R−S, (**c**) R−SW, (**d**) S−R, (**e**) S−S, (**f**) S−SW, (**g**) SW−R, (**h**) SW−S, and (**i**) SW−SW.

In this section, an analysis of the combinations between inner and outer topologies is presented in order to reveal which of the analyzed cases achieves outer synchronization in the most optimal way, i.e., with the minimum values of coupling strength $c_{1}$ and $c_{2}$. As a notation to refer to the different inner–outer coupling network topologies, we use the letter *R* for ring, *S* for star, and SW for small-world networks, for example, for a combination of the inner ring and outer ring topologies, we denoted it with notation R−R (see Figure 1b), for a combination of inner star and outer star topologies, we denoted it with notation S−S (see Figure 2b), for a combination of inner small-world and outer small-world topologies, we denoted it with notation SW−SW (see Figure 3b), and so on for the other inner–outer coupling network topologies. On the other hand, we can have some of the combinations of inner–outer coupling network topologies where we have previously established that a suitable option to use is $\Gamma_{1,0,1}=diag\left[1,0,1\right]$, with this in mind, we can perform an analysis by applying different values of $\Gamma_{o}$ to verify which case performs better for outer synchronization. Figure 7 shows the maximum Lyapunov exponents $\lambda_{max}$ setting the value $c_{1}=2$ for different values of $\Gamma_{o}$, (as previously mentioned, $\Gamma_{o}$ is the matrix that indicates which nodes are chosen to outwardly couple the networks), the most representative values are chosen (for a range $0\leq c_{2}\leq 5$) as follows; $\Gamma_{o1}=diag\left[1,0,0,0,0\right]$, $\Gamma_{o2}=diag\left[1,1,0,0,0\right]$, $\Gamma_{o3}=diag\left[1,1,1,0,0\right]$, $\Gamma_{o4}=diag\left[1,1,1,1,0\right]$, and $\Gamma_{o5}=diag\left[1,1,1,1,1\right]$. Perhaps one could think that the more outer couplings, the better performance when it comes to achieving outer synchronization, but as we can see from Figure 7, in all cases, for the lower bounds of values $c_{2}$ there is no difference in selecting some $\Gamma_{o}$ unless redundancy is wanted in the outer couplings among the networks, even for the upper bounds $c_{2}$, using fewer nodes to outwardly couple the networks results in a better performance to achieve outer synchronization, so for practical purposes, in the following, we use $\Gamma_{o1}=diag\left[1,0,0,0,0\right]$.

**Figure 8:**
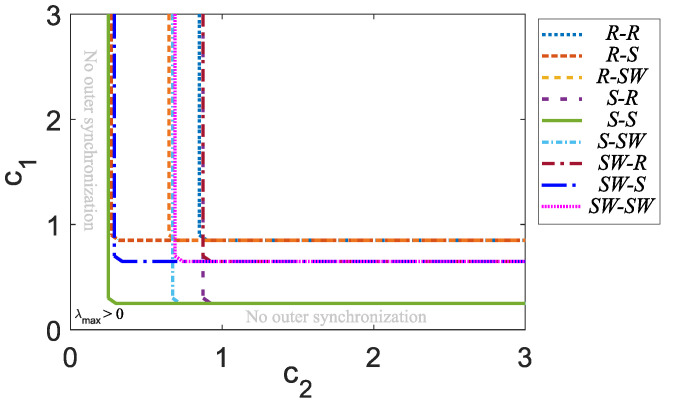
Maximum Lyapunov exponent $\lambda_{max}$ for $c_{1}$ versus $c_{2}$ applying different inner–outer topologies with Γ=diag[1,0,1] and $\Gamma_{o}=diag\left[1,0,0,0,0\right]$.

In order to analyze the outer synchronization state using different inner–outer coupling network topologies, that is, for R−R, R−S, R−SW, S−R, S−S, S−SW, SW−R, SW−S, and SW−SW, we calculate the maximum Lyapunov exponent $\lambda_{max}$, taking into account $c_{1}$ versus $c_{2}$ with N=5, M=5, Γ=diag[1,0,1], and $\Gamma_{o}=diag\left[1,0,0,0,0\right]$, where Figure 8 shows that the best combination in order to achieve outer synchronization in a master–slave configuration is an S−S coupling network topology, which encompasses all other inner–outer coupling network topologies.

### 5.3. A Big Network in Inner–Outer Network Coupling Topology S−S in Master–Slave Configuration

**Figure 9:**
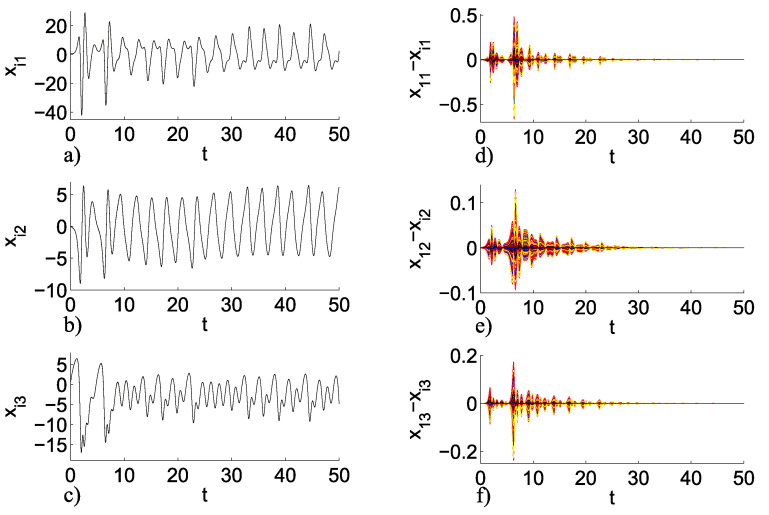
Inner–outer coupling network topology S−S with N=5 and M=100. Temporal dynamics (**a**) $x_{i1}$, (**b**) $x_{i2}$, and (**c**) $x_{i3}$, and errors between the master node and the other nodes in the network; (**d**) $x_{11}-x_{i1}$, (**e**) $x_{12}-x_{i2}$, and (**f**) $x_{13}-x_{i3}$.

Based on the obtained results of the Section 5.2, an inner–outer network coupling topology S−S is now used (the most suitable to achieve outer synchronization in a master–slave configuration) for N=5 nodes and a large number of networks M=100 with Γ=diag[1,0,1], $\Gamma_{o}=diag\left[1,0,0,0,0\right]$, $A_{i}=A_{\mathrm{is}}$ as in (11), and $A_{o}\left(M\times M\right)$ selected as follows

(13) $$A_{o}=\left[\begin{array}{ccccc} 0 & 0 & 0 & \ldots & 0\\ 1 & -1 & 0 & \ldots & 0\\ 1 & 0 & -1 & \ldots & 0\\ \vdots & \ddots & \ddots & \ddots & \vdots \\ 1 & 0 & 0 & \ldots & -1 \end{array}\right],$$

where we can deduce that the maximum Lyapunov exponent $\lambda_{max}$ (for this case it is the same as the case of Figure 7e), where to achieve outer synchronization we can either use $c_{1}=2$ or $c_{2}=2$. It should be noted that this analysis can be extended for any other chaotic node, number of nodes *N*, and number of networks *M*. Figure 9a–c shows the temporal dynamics for this case, and Figure 9d–f shows the errors between the master node and the other nodes in the network, the values of the initial conditions are taken as follows $x_{i1,i2,i3}\left(0\right)=\left[rand\left(1.01,1.02\right),0.1,0.1\right]$ for i=1,2,…,N×M.

Figure 9 corroborates that this analysis can be extended for a large number of networks *M* and, therefore, a large number of nodes N×M.

## 6. Conclusions

By means of an analysis based on the master stability function approach, which is widely used to determine synchronization in networks, it has been shown that if we use Γ=diag[1,0,1] in inner coupling topologies *R*, *S*, and SW, we obtain a suitable stable inner synchronization state; therefore, these two states ($x_{i,1}$ and $x_{i,3}$ from (9)) were used as the suitable option to connect the nodes innerly and outwardly. On the other hand, there is not much difference when we outwardly couple networks with more than one node using inner–outer coupling network topologies R−R, R−S, R−SW, S−R, S−S, S−SW, SW−R, SW−S, and SW−SW, so it was determined to use a $\Gamma_{o}=diag\left[1,0,0,0,0\right]$. The final analysis shows us that the best combination to achieve outer synchronization in a master–slave configuration is the inner–outer coupling network topology S−S. Additionally, an example for a large number of networks was presented using the inner–outer coupling network topology S−S as an example to verify the obtained results from the accomplished analysis. Moreover, we can deduce that some inner–outer coupling topologies will be better than others for some potential applications despite the fact that the inner–outer coupling network topology S−S achieves outer synchronization more efficiently. On the other hand, the possibility of extending this study is open, for example, we can use different configurations of matrix A, different forms, and eventually different dimensions of the chaotic node; also, we can increase the number of nodes *N* and the number of networks *M* in order to combine this study with other synchronization control techniques, such as, for example, the fractal variational principle for an optimal control problem and the synchronization in the fractal vibration systems, or the possibility of making some transformation to a fractional order study, among others.

## Data Availability

The data used to support the findings of this study are included within the article.
